# Smallholder farmers’ knowledge, attitudes, and practices (KAP) regarding agricultural inputs with a focus on agricultural biologicals

**DOI:** 10.1016/j.heliyon.2024.e26719

**Published:** 2024-02-20

**Authors:** Tewodros Mulugeta, Mesia Ilomo, Allan Mueke, Cecillia Onyango, Lerato Matsaunyane, Quenton Kritzinger, Erik Alexandersson

**Affiliations:** a Department of Biology, College of Natural and Computational Science, Kotebe University of Education, Addis Ababa, Ethiopia; b Department of Plant and Soil Science, University of Pretoria, Pretoria, South Africa; c Department of Animal Health and Production, School of Pure and Applied Science, Mount Kenya University, General Kago Rd PO BOX 342-01000, Thika, Kenya; d Department of Plant Science and Crop Protection, University of Nairobi, Nairobi, Kenya; e Department of Plant Breeding, Agricultural Research Council-Vegetable and Ornamental Plants, Pretoria, 0001, South Africa; f Department of Plant Breeding, Swedish University of Agricultural Sciences (SLU), SE-23053, Lomma, Sweden

**Keywords:** Agricultural inputs, Biologicals, Biostimulants, Biofertilizers, Biopesticides, Knowledge attitudes and practices (KAP), Sub-Saharan Africa, Smallholder farmers

## Abstract

There is a general drive to reduce pesticide use owing to the potential negative effects of pesticides on the environment and human health. The EU Commission, for example, through its “Farm to Fork Strategy,” has proposed to decrease the use of hazardous chemical pesticides by 50% by 2030. In addition, smallholder farmers in low-income countries do not always follow pesticide safety precautions. This necessitates the introduction of low-risk crop protection strategies also suited for these farmers. Agricultural biologicals can substitute for, or at least partially replace hazardous chemical pesticides. While the market for and use of biologicals is growing quickly in industrialized countries, this practice remains limited in sub-Saharan Africa. To understand the reason behind the low adoption of biologicals, this study examined the knowledge, attitudes, and practices toward biologicals among 150 smallholder farmers in the Chole district in Ethiopia. All farmers used chemical pesticides and/or inorganic fertilizers to protect crops, improve yields, and comply with government regulations. The use of biologicals was, however, restricted to one group of biologicals, bio-fertilizers, which approximately 60% of farmers used, and no use of biologicals for plant protection was reported. Even though the understanding of the concept of biologicals was deemed high among respondents, the majority (90%) did not identify biologicals as safer alternatives to conventional agricultural inputs. More than half of the respondents (54%) did not recommend biologicals as safer alternatives to their colleagues. Nevertheless, even if the responding farmers did not perceive biologicals as risk-free, they had a positive attitude towards biologicals when it came to producing healthy food and increasing crop yields and incomes. In comparison to the positive attitude, farmers’ knowledge and practice of biologicals were generally low; thus, efforts are needed to create awareness among farmers.

## 1 Introduction

Agricultural biologicals is a broad term encompassing living organisms and their derivatives applied in farming systems serving as biostimulants, biocontrol agents, resistance inducers, or biofertilizers offer promising solutions. Biologicals have the potential to enhance crop performance, increase yield and reduce susceptibility to biotic and abiotic factors [1,2]. If appropriately introduced, they can aid the development of ecologically, socially, and economically sustainable agricultural practices, reducing the reliance on nonrenewable resources. Moreover, they can align with the increasing demand for organic products and sustainable farming [3]. Thus, there is a burgeoning interest among industry, suppliers, and policy-makers in adapting biologicals as alternatives to conventional agricultural chemicals and inorganic fertilizers [4,5].

The market for agricultural biologicals is rapidly growing. Notably, plant biostimulants alone have an annual world market growth of 14% and were estimated to have worldwide reached USD 3.7 billion in 2022 [6,7]. The estimated market share for biostimulants in the Middle East and Africa was a mere USD 70 million in 2016 [8], which is in stark contrast to Europe, where the value was estimated to be over USD 900 million [9]. It should, however, be noted that reliable data on the annual use of biologicals in Sub-Saharan Africa (SSA) is missing.

Despite their potential, low-income countries witness a limited adoption of biologicals compared to the industrialized world. One reason is that a large proportion of smallholder farmers in sub-Saharan Africa have not adapted biologicals. In Ethiopia, for instance, smallholders constitute the majority of the workforce with approximately 80% of the population engaged in agriculture. The sector is the backbone of the Ethiopian economy, contributing approximately 50% of Ethiopia's gross domestic product (GDP) [10,11]. It makes smallholder farmers key actors when it comes to the adaption of biologicals in Ethiopia and countries with a similarly structured agricultural sector.

There are several factors underlying the low adoption of biologicals in African agriculture. Smallholder farmers face additional challenges in utilizing biologicals including concerns about effectiveness, speed of action, spectrum of activity, stability, availability, and affordability [12,13]. Furthermore, regulations to produce, import, register, pack and distribute biologicals are not adequately framed into policies and guidelines [10,11]. For instance, Ethiopia has a pesticide registration and control proclamation (674/210), which regulates pesticides. However, the proclamation lacks information about biologicals. Today, there is a large variation in requirements for registration of biologicals between countries [14]. Finally, research conducted has predominantly been limited to *in vitro* experiments, and the lack of field verification in African conditions hampers the necessary lab-to-field transaction and reduces the practical use of biologicals [3,15,16].

The KAP theory underscores that altering human behavior occurs in three consecutive steps: acquiring accurate knowledge, cultivating supportive attitudes, and adopting corresponding behaviors or practices [[17], [18], [19]]. This sequence fosters behavioral change regarding the use of biologicals in agriculture. A better understanding of smallholder farmers’ knowledge, attitudes, and practices regarding agricultural inputs is necessary to increase the sustainability of the agriculture sector and reshape public policies to better accommodate biologicals in the African agrosystem. To investigate the underutilization of biologicals in low-income countries, a comprehensive examination of smallholder farmers' KAP concerning agricultural inputs, focusing on commercially available biologicals was conducted in Ethiopia. To the best of our knowledge, this is the first KAP analysis regarding the use of biologicals in Ethiopia.

## 2 Materials and methods

### 2.1 The study site

**Fig. 1:**
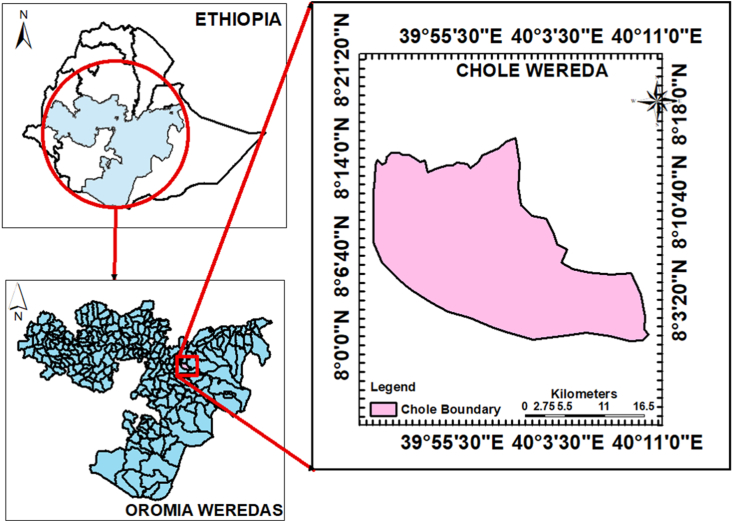
Map of the study site.

The study was conducted in the Chole district, which is located in the Arsi zone of the Oromia National Regional State in South-Eastern Ethiopia (Fig. 1). The sampled villages are located at an elevation of ∼2700 m above sea level. The Chole district covers an area of 766 km^2^. The climate is temperate, with day temperatures ranging from 15 to 25 °C. The mean annual rainfall is 1000 mm. Subsistence farmers typically make a living through mixed crop-livestock agriculture.

### 2.2 Methods of data collection

In January 2022, face-to-face interviews were conducted with 150 farmers to document their knowledge, attitudes and practices (KAP) regarding inputs with a focus on biologicals in the agricultural system. Respondents were selected with a field-based approach while walking between sparsely scattered houses in rural areas, and interviews were also conducted with farmers encountered while working in their fields. The interviews were conducted with the participants' consent, and they were informed of their right to terminate the interview at any time. Structured interview questions were used, and the questionnaire's validity was confirmed through an initial test involving ten farmers.

The questionnaire was prepared in Amharic, one of the locally spoken languages. The questions were divided into five major parts: general demography, agriculture, and farming practice, use of agricultural inputs, knowledge and use of biologicals, and attitude to biologicals, containing 9, 10, 19, 24, and 15 questions, respectively. The questionnaire consisted of closed-ended, open-ended, dichotomous, and multiple-choice questions. The attitude questions were rated on a Likert scale from 1 to 5 to rank respondents’ attitudes: strongly disagree, disagree, undecided, agree, and strongly agree. Knowledge, attitude, and practice were calculated by taking the mean of the sum of scores for each KAP question as a good or poor category. Nine, 15, and eight questions were categorized as related to knowledge, attitude, and practice, respectively.

## 3 Data analysis

The data were entered into Microsoft Excel 2010, cross-checked, and exported into JMP Pro 17 for analysis. For dichotomous questions, the value “1” was given to responses considered in favour of using agri-inputs/biological, whereas the reverse was given “0”. For non-dichotomous open-ended and close-ended questions, responses that were deemed adequate, desirable or appropriate with regard to KAP were assigned a value of 1, whereas responses deemed the opposite were assigned a “0” value. The questions and the scoring of the answers to each question in given in the Supplementary material file “coding KAP 0 and 1”. The aggregate of each participant's responses for each category is tallied. The internal consistency of the data was assessed using a multivariate Cronbach's alpha reliability test. The continuous dataset was summarized by descriptive statistics such as mean, median, standard deviation, and range. Percentages and frequency tables were used for categorical data. The association between demographic and socio-economic aspects (age, sex, education, training, and farm size) and farmers' knowledge, attitudes, and practices of agricultural inputs and biologicals was analyzed using the Pearson chi-square test. Each demographic characteristic was associated with aggregate responses to the knowledge, attitude, and practice questions. In addition, multivariate logistic regression analysis was conducted to assess the association between the dependent and independent variables and identify statistically significant results. The relationship between each pair of respondents' KAP was analyzed using the nonparametric Spearman's rho rank correlation test with responses coded as 0 and 1 within each aspect of knowledge, attitude, and practice.

## 4 Results

### 4.1 Socio-demographic characteristics of the respondents

As shown in Table 1, 96.7% (145) were male and 3.3 % (5) were female respondents. The mean age was 50.2 years, ranging between 20 and 74 years. Most of the respondents were married (97.3%), and the average family size was six, ranging between 1 and 18 members. The mean number of male and female family members was 3.3 and 2.7, respectively, ranging between 1 and 15 males and 0 and 12 females. About a third (34.7%) of the respondents had completed primary school. Income calculations were based on 134 out of the 150 respondents since 16 were unable to estimate incomes. The mean annual family income stood at USD 565.5 (ETB 29,397). Almost all respondents (98.7%) relied solely on agriculture for their livelihood, with only two respondents had side jobs.

**Table 1:** The socio-demographic characteristics of the respondents are depicted in numeric values and percentages in brackets (Number of respondents = 150).

**Sex**	
Male	145 (96.7)
Female	5 (3.3)
**Marital status**	
Married	146 (97.3)
Single	3 (2)
Widowed	1 (0.7)
**Livelihood**	
Farming	148 (98.7)
Farming + side jobs	2 (1.3)
**Educational background**	
No education	43 (28.7)
Non-formal/church	16 (10.7)
Primary	52 (34.7)
Secondary	33 (22.0)
College Diploma	6 (4.0)
**Head of household**	
Male	145 (96.7)
Female	5 (3.3)

N(%) = Count and percentage.

### 4.2 Agriculture and farming practice

The respondents’ farming experience spanned from 2 to 50 years, with an average of 27.3 years. The largest farmland reported was 3.75 ha, with an average landholding size of 1.13 ha. Only four (2.7%) of the respondents rented land and 146 (97.3%) owned land. Among landowners, 44 out of 146 (30.1%) respondents rented an additional piece of land.

All farmers were involved in crop production, cultivating primarily wheat, maize and teff in the main rainy season, while barley was the exclusive short rainy season crop grown by all the respondents (Table 2). None of the respondents used irrigation for crop production. More than half of the respondents (52.7%) relied on labor from family, relatives, and friends during the previous production season (Table 2). Four respondents (2.7%) could not exactly quantify their production from the last harvest. For the remaining 146 (97.3%), the average yield was 2.6 tons ranging between 0.3 and 9.0 tons. More than three-fourths of the respondents (79.3%) sold part of their produce at the local market. Fifty-eight (38.7%) of the respondents were unable to categorize themselves as organic or inorganic farmers, whereas the remaining 45 (30.0%) and 47 (31.3%) claimed themselves as either non-organic or organic farmers.

**Table 2:** Summary of farmer responses on labor sources, main rainy season crop, produce selling practices, and organic farming status (N = 150, numeric values and percentages.

**What labor source did you use last season?**	
Family	5 (3.3)
Family, Friends	3 (2.0)
Family, Paid	12 (8.0)
Family, Paid, Friends	7 (4.7)
Family, Paid, Relative	1 (0.7)
Family, Paid, Relatives, Friends	31 (20.7)
Family, Relatives, Friends	79 (52.7)
Family, Relative	4 (2.7)
Paid	2 (1.3)
Paid, Friends	5 (3.3)
Relative	1 (0.7)
**Main rainy season crop**	
Wheat	1 (0.67)
Wheat, Teff	69 (46.0)
Wheat, Maize	80 (53.3)
**Do you sell your produce?**	
No	31 (20.7)
Yes	119 (79.3)
**Are you an organic farmer?**	
I do not know	58 (38.7)
No	45 (30.0)
Yes	47 (31.3)

N (%) = Count and percentage.

### 4.3 The practice of agricultural inputs

Cronbach's alpha values of 0.87, 0.83, and 0.71 were obtained for the knowledge, attitude, and practice category aggregate, respectively. The overall Cronbach's alpha value for the entire set was 0.86. According to Tavakol et al. [20], alpha values greater than 0.7 and less than 0.9 are acceptable; thus, the internal consistency of the current data is good.

**Fig. 2:**
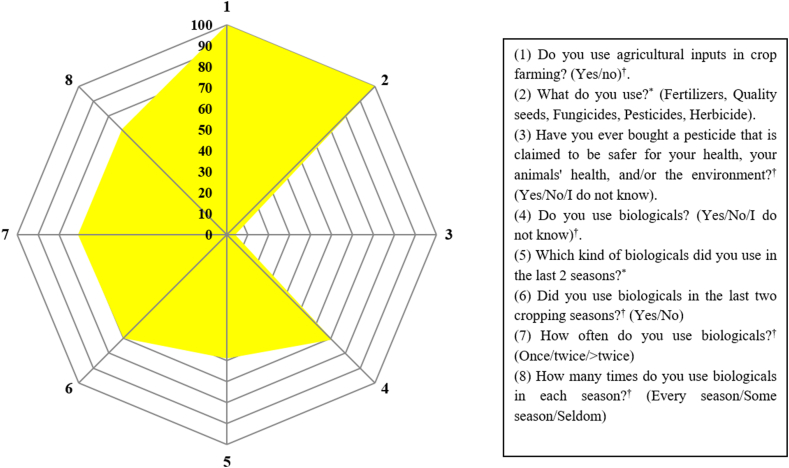
Radar chart illustrating farmers' practices of using agricultural inputs, including biologicals (N = 150, data presented in percentages). Open-ended questions are indicated by “*”, and close-ended questions are indicated by “^†^”. In question number 3 and 4, ‘No’ responses and ‘I do not know’ have been treated as ‘No’ for data analysis and presentation, representing inappropriate practice.” The yellow shape represents the proportion of the responses depicting practices deemed appropriate. For question five, an appropriate practice includes any mention of commercially available biological products. Regarding questions 7 and 8, all responses are considered appropriate practices as they signify the usage of biologicals, regardless of frequency.

Eight questions were employed to evaluate the respondents’ practices of agricultural inputs including biologicals (Fig. 2). All respondent farmers used one or a few agricultural inputs such as fertilizers, certified seeds, herbicides, fungicides, or insecticides. Calcium carbonate, manure, and compost were also used by farmers, but none of these were taken into account as biologicals in the context of this study since only commercially available biologicals were considered. The primary agricultural inputs used by the majority (131; 87.0%) of the respondents were fertilizers, quality seeds, fungicides, pesticides, and herbicides. Most of the farmers 110 (73.3%) relied on the recommendations by extension service officers and retailers for pesticide usage, supplemented by advice from neighbors. Most of the farmers (96.0%) reported they never bought pesticides marked as safer for human and animal health, and/or the environment. Despite 71.3 % (107/150) of the respondents claimed to use biologicals, based on the examples they provided, only 12.0% (18/107) were found to be using biologicals. Furthermore, 23.3% were uncertain about their usage, while the remaining 5.3% cited high price (50.0%) or inaccessibility (50.0%) as a reason for never using biologicals. Regarding the kind of biologicals farmers used in the last cropping season, 59% mentioned biofertilizers as representative of biologicals. Apart from biofertilizers, only one farmer claimed used biopesticides. The majority (71%) of the respondents reported they used biologicals in all seasons and once or more per season.

### 4.4 Knowledge of biologicals as agricultural inputs

**Fig. 3:**
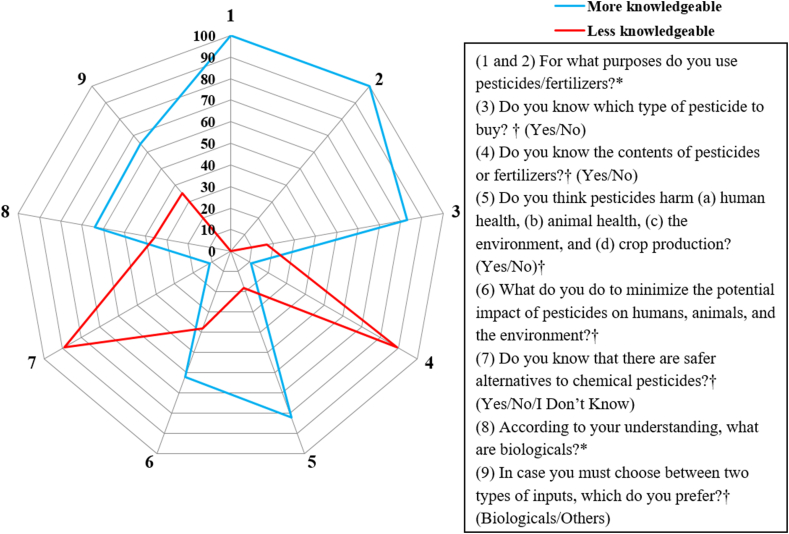
Radar chart illustrating farmers' knowledge about conventional agricultural inputs and biologicals (N = 150, data presented in percentages). Open-ended questions are indicated by “*”, and close-ended questions are indicated by “†”. More knowledgeable responses to questions 1 and 2 include aiming to reduce crop loss, meet market demand, and enhance production. In question 6, usage of personal protective equipment, buying safer pesticides, and seeking advice from extension officers are deemed knowledgeable whereas I do nothing and I do not know make less knowledgeable responses. In question 8, a knowledgeable response involves providing an example of a biological product.

Farmers responded to nine knowledge-based questions concerning general agricultural inputs and biologicals (Fig. 3). Based on the responses, all the respondents provided insights into their motivations for using pesticides and fertilizers. Farmers commonly cited using pesticides to meet market demand and reduce crop loss. Reduction of crop loss was the key reason why the majority of the respondents (96.0%) used pesticides. Fertilizer usage for most of the farmers was to improve production (98.7%), and government regulation was also raised by a farmer. Most of the farmers were certain about the pesticide they should buy (83%), and the rest (17.0%) did not know the type of pesticide to buy.

Farmers exhibited limited knowledge about the content or active ingredient of purchased fertilizers and pesticides, with 89.0% unaware of their specific composition. A large proportion of the farmers (82.0%) reported that pesticides may negatively affect human and animal health, the environment, or crops. Almost three-fourths (71.3%) of the farmers claimed that pesticides harm human health, the environment, animals, and crop production. Eighteen percent were aware of pesticide impact on human and animal health. Besides, 10.0% were not aware of the impact of pesticides on crop production, and only one respondent disagreed that pesticides influence crop production. More than 60% of the farmers adopted used one or more suitable measures to minimize pesticide impact such as utilizing personal protective equipment, purchasing safer pesticides, and seeking advice from extension officers, whereas, close to 40% were deemed less knowledgeable regarding these mitigation strategies. The farmers’ awareness of safer alternatives to conventional pesticides and fertilizers was low, with 90% of farmers indicating no knowledge of such alternatives. Nevertheless, over 60% demonstrated an understanding of biologicals based on the examples they provided. Moreover, the majority (65%) expressed a preference for biologicals over conventional pesticides and fertilizers.

### 4.5 Attitudes of farmers on biologicals as agricultural inputs

**Fig. 4:**
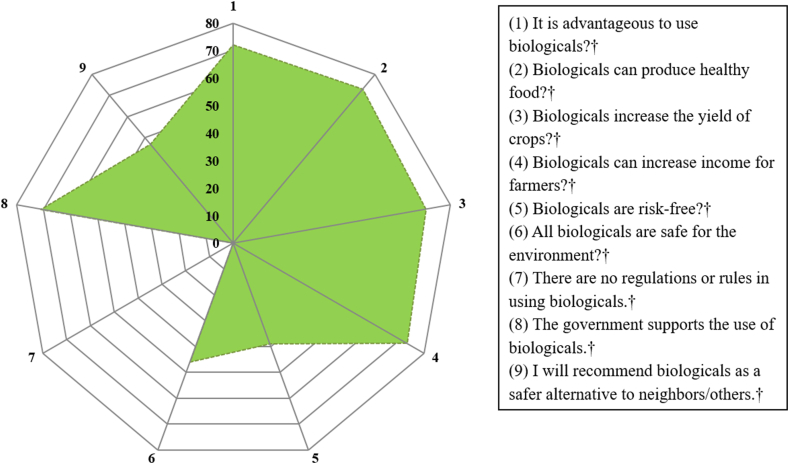
Radar chart illustrating farmers' attitudes about biologicals (N = 150, data presented in percentages). The responses indicated below were coded into negative or positive attitudes to biologicals to produce the chart. ^†^close-ended questions. The light green shape represents the proportion of the responses depicting positive attitudes. All the questions are on a Likert scale with five alternatives -Strongly disagree, Disagree, Undecided, Agree, and Strongly Agree.

The attitude of smallholder farmers towards biologicals was assessed through a series of nine questions. Most of the respondents (72%) recognized the benefits of using biologicals to produce healthy food (73%), increase the yield of crops (71%), and improve farmers’ income, (73%). Nevertheless, more than half (54%) of the respondents would not recommend biologicals as a safer alternative to others. About 71% of the farmers expressed the need for governmental support in promoting the use of biologicals. Most of the farmers (61%) disagreed with the statement “Biologicals are risk-free,” and more than half (54%) of them also disagreed that biologicals are safe for the environment. Similarly, all the respondents disagreed about the presence of regulations governing the usage of biologicals within the country (Fig. 4).

### 4.6 Association analysis

The Pearson chi-square analysis revealed significant associations between farmers' knowledge about agricultural inputs, particularly biologicals, and factors such as age, farm size, and training. Most of the farmers within the age group 41 to 50 and >50 were more knowledgeable about agricultural inputs and biologicals. Moreover, larger farms correlated with less knowledge level about agricultural inputs and biologicals. Furthermore, farmers’ age, gender, educational background, and farm size significantly influenced attitudes and practices toward the use of agricultural inputs and biologicals (Table 3).

**Table 3:** Pearson chi-square analysis of the association between demographic and socio-economic characteristics and KAP (Knowledge, Attitudes, and Practices) towards agricultural inputs and biologicals. The values in the table depict the number of respondents in each category and the values in the bracket show the percentage from the whole. (N = 150, data presented in counts and percentages).

Characteristics	Category	Knowledge		Attitude		Practice	
		More	Less	Positive	Negative	More appropriate	Less appropriate
Age	18 to 30	4 (2.4%)	2 (1.6%)	2 (1.6%)	4 (2.4%)	3 (2.05%)	3 (2.05%)
	31 to 40	13 (8.4%)	5 (3.6%)	12 (7.8%)	6 (4.2%)	14 (9.5%)	4 (2.5%)
	41 to 50	41 (27.3%)	18 (12.1%)	35 (23.2%)	24 (16.1%)	41 (27.5%)	18 (11.8%)
	>50	39 (26.1%)	28 (18.6%)	33 (22.1%)	34 (22.5%)	44 (29.2%)	23 (15.5%)
***χ***^***2***^***value***		18.2		22.4		14	
***P value***		0.0004*		0.0001*		0.003*	
Sex	Male	93 (62.1%)	52 (34.6%)	81 (53.8%)	64 (42.9%)	100 (66.8%)	45 (29.8%)
	Female	3 (2.1%)	2 (1.3%)	1 (0.9%)	4 (2.4%)	2 (1.6%)	3 (1.8%)
***χ***^***2***^***value***		0.1		14.7		8.4	
***P value***		0.78		0.0001*		0.004*	
Education	No education	26 (17.5%)	17 (11.2%)	21 (14%)	22 (14.7%)	27 (18.3%)	16 (10.3%)
	Non-formal	11 (7.1%)	5 (3.6%)	12 (7.8%)	4 (2.9%)	12 (8.3%)	4 (2.4%)
	Primary	33 (22.1%)	19 (12.6%)	26 (17.3%)	26 (17.3%)	35 (23.1%)	17 (11.6%)
	Secondary	22 (15%)	11 (7%)	20 (13.3%)	13 (8.7%)	24 (16.1%)	9 (5.9%)
	Diploma	4 (2.5%)	2 (1.5%)	3 (2%)	3 (2%)	4 (2.7%)	2 (1.3%)
***χ***^***2***^***value***		4.1		33		11.3	
***P value***		0.4		0.0001*		0.02*	
Farm size	<1	48 (32.1%)	22 (14.6%)	41 (27.6%)	29 (19.1%)	51 (33.8%)	19 (12.8%)
	1 to 2	42 (27.7%)	27 (18.3%)	35 (23.6%)	34 (22.4%)	45 (29.8%)	24 (16.2%)
	>2	7 (4.4%)	4 (3%)	5 (3.5%)	6 (3.9%)	7 (4.8%)	4 (2.6%)
***χ***^***2***^***value***		10.8		9.7		8.1	
***P value***		0.005*		0.01*		0.02*	
Training	Trained	1 (0.7%)	0 (0%)	1 (0.7%)	0 (0%)	1 (0.7%)	0 (0%)
	Untrained	95 (63.5%)	54 (35.9%)	81 (54.1%)	68 (45.2%)	102 (67.8%)	47 (31.5%)
***χ***^***2***^***value***		5.1		2		3	
***P value***		0.02*		0.2		0.1	

The likelihood ratio of the multivariate logistic regression analysis showed that age and training significantly affected the respondents’ knowledge of biologicals and utilization of agricultural inputs (p = 0.0002 and p = 0.002, respectively). Compared to farmers who were in the age group >50, the age groups 31 to 40 and 41 to 50 were 2.1 (AOR = 2.05, 95% CIs: 1.30–3.23) and 1.8 (AOR = 1.83, 95% CIs: 1.371–2.234) times more likely to be more knowledgeable about biologicals.

Age and educational background significantly affect the farmers’ attitudes toward agricultural inputs and biologicals. Compared to the youngest age group (18–30), the older age groups 31 to 40, 41 to 50, and >50 were found to be 3.8 times (AOR = 3.79, 95% CIs: 1.925–7.456), 3.4 times (AOR = 3.36, 95% CIs: 1.808–6.232), and 2.0 times (AOR = 1.99, 95% CIs: 1.059–3.752) more likely to have a positive attitude regarding agricultural inputs-biologicals, respectively. Compared to illiterate farmers, farmers who had informal or church and secondary education were 2.7 times (AOR = 2.69, 95% CIs: 1.764–4.128) and 1.5 times (AOR = 1.49, 95% CIs: 1.054–2.118) more likely to have a positive attitude towards agricultural inputs and biologicals, respectively.

Similarly, age and educational background significantly affected farmers' utilization of agricultural inputs. The results further revealed that the respondents in the age groups 31 to 40, 41 to 50, and >50 years had a practical experience of usage (AOR = 3.67, 95% CIs: 1.732, 7.784; AOR = 2.475, 95% CIs: 1.273, 4.809; and AOR = 1.83, 95% CIs: 0.924, 3.622, respectively) compared with the farmers who were in the 18 to 30 age group. Furthermore, farmers who attended informal or church school and secondary education were 1.8 times (AOR = 1.79, 95% CIs: 1.112, 2.872) and 1.4 times (OR = 1.40, 95% CIs: 0.931, 2.106) more likely to practice agricultural inputs-biological-utilization, respectively, than the illiterate farmers. Respondents’ knowledge positively and significantly correlated with attitude and practice and attitude significantly and positively correlated with practice (Table 4).

**Table 4:** Correlation between farmers’ knowledge, attitude, and practice towards utilization of agricultural inputs and biologicals. This table illustrates the correlation among farmers' Knowledge (K), Attitude (A), and Practice (P) concerning the adoption and utilization of agricultural inputs and biologicals. The analysis used responses coded as 0 and 1 within each aspect of Knowledge, Attitude, and Practice (KAP).

Correlation	Spearman's ρ (*Prob* > *|ρ)*
K-A	0.35 (<0.0001)
K–P	0.124 (<0.0001)
A-P	0.32 (<0.0001)

## 5 Discussion

This study investigates smallholder farmers' knowledge, attitudes, and practices on the use of agricultural inputs, particularly biologicals. It is worth noting that the participation of female respondents was notably low, a reflection of the prevailing norm in many regions of Ethiopia, where agricultural asset ownership typically rests with men, except in cases of female-headed households from circumstances like divorce or widowhood [[21], [22], [23]]. To obtain women farmers' insights, it will be necessary to specifically target women-headed households.

Most of the farmers in this study had adequate knowledge about the purpose of utilizing agricultural input in farming practices. Enhancing crop production emerged as the key reason behind fertilizer usage among respondents (Fig. 3). Farmers used pesticides to reduce crop loss (96%) and fulfill market demand, which is similar to findings among farmers in Nepal [24] and smallholder farmers in Kenya where 87% of farmers used chemical pesticides to control crop pests [12]. More than 80% of the farmers reported to have known what type of pesticide or fertilizer to buy. This is considerably higher than reported in a study conducted in Ethiopia by (Ocho et al.) [25], in which 48% of farmers were unaware of which type of pesticide they used. However, only 11% of the respondents in the present study demonstrated awareness regarding the content of the pesticides and fertilizers they used.

Pesticides pose potential risks to human health, the environment, and non-target organisms, [26]. 82% of the farmers acknowledged the possible negative impact of pesticides and fertilizers on human and animal health, and the environment. This is consistent with previous findings [[27], [28], [29], [30]]. Despite their awareness of these potential negative impacts associated with conventional chemical agricultural inputs, farmers continued widespread use, due to efficiency and affordability. Similarly, smallholder farmers in Kenya recognized the adverse impacts of chemical pesticides on health and the environment; however, compared to chemical pesticides, biopesticides were perceived as less favorable due to concerns regarding their effectiveness, speed of action, the spectrum of activity, availability, and affordability [12].

Our study reveals that farmers do not associate safety with biologicals. Despite 62% of the farmers being deemed knowledgeable about biologicals, only 10% acknowledged awareness of the existence of safer alternatives. Moreover, 60% of the respondents did not view biologicals as consistently risk-free. Safety consideration scarcely factored into farmers’ decision-making when purchasing agricultural inputs.

Negatu et al. [31], reported that farmers had limited knowledge regarding alternatives to chemical pesticides such as biopesticides, cultural methods, and IPM, with only 31% of the respondents being able to mention at least one alternative. Alternatives to chemical pesticides were more frequently cited by large-scale greenhouse workers. Similarly, in Egypt, a study reported that 79% of farmers lacked awareness of available alternatives to chemical pesticides for pest management [32]. A similar study reported that less than 20% of the farmers had heard about biopesticides [29].

Among surveyed farmers, 65% expressed a preference for biologicals over conventional pesticides and fertilizers. While the use of biopesticides was reported in large-scale greenhouses and large-scale farms [31], this study's farmers solely utilized biofertilizers. Based on the product farmers named during the interview, we deemed 59% of the farmers to have had a practice of using biologicals in the form of biofertilizers, however, this was lower than what farmers claimed (71%). Such disparity might stem from farmers' misunderstanding of the concept of biologicals. Similarly, prior studies have highlighted instances where products identified as biopesticides by farmers did not align with actual biopesticides [12].

In this survey, 72% of the farmers recognized the advantages of employing biologicals in agriculture. Nearly three-fourths of the farmers agreed on the beneficial role of biologicals in the production of healthy food, increasing the yield of crops, and enhancing farmers’ income. Likewise, the benefits of organic agriculture are associated with environmental and food safety, as well as human and animal health [33].

Despite the farmers’ generally positive attitude and good awareness of biologicals, it is surprising that more than half of the farmers did not recommend biologicals as a safer alternative (Fig. 4). Similar observations have been reported where trained personnel do not regularly recommend biopesticides [34]. Recommendation to use biologicals by experts or farmers might depend on factors such as accessibility, affordability, efficiency, ease of storage and application.

The factors affecting farmers' knowledge about agricultural inputs particularly biologicals were age, farm size, and level of training (Table 3). Older respondents ->40 years-exhibited greater familiarity with agricultural inputs than the rest. Age is known to play a significant role in dictating farmers' decision to use agricultural inputs or the adoption of agricultural technology [[35], [36], [37], [38]]. Trained farmers were more knowledgeable about agricultural inputs and biologicals than untrained. This is in line with previous studies showing that farmers’ knowledge regarding pesticide safety and hazard control was positively correlated to the training they had had. Trained farmers also showed high safety behavior [[39], [40], [41], [42]]. Unexpectedly, farmers who used a larger piece of land had less knowledge about agricultural inputs and biologicals.

Agricultural input and biological utilization practice were significantly affected by age and educational background. The age groups 31 to 40, 41 to 50, and >50 years were deemed to have a better practice than farmers who were in the age group 18 to 30. This is contrary to a study, which found that older Chinese rice farmers had a lower probability of adopting biopesticides [43]. The knowledge–attitudes, knowledge–practices, and attitudes–practices pairs were significantly positively correlated (Table 4). A KAP study of smallholder farmers in Kenya and Uganda showed that biological usage for pest management was lower compared to farmers’ knowledge [44]. In line with several previous reports, this study found that farmers who had informal or church and secondary education had significantly higher knowledge and positive attitudes regarding agricultural inputs and biologicals than illiterate farmers [[45], [46], [47], [48], [49], [50]]. Thus, both the availability of information and the ability to apprehend it, are likely to play roles in the adaption of biologicals.

## 6 Conclusion

This study assessed smallholder farmers’ knowledge, attitude, and practice of agri-inputs focusing on biologicals as alternatives to conventional chemicals. Farmers were judged to have appropriate practices of using agricultural inputs; however, the practice of biological use was limited to bio-fertilizers. Even if most of the farmers were certain about the pesticide to buy, knowledge about the content was limited. Farmers were, however, aware of the possible negative impacts of conventional chemicals on human and animal health and the environment. Even if most of the respondents perceived biologicals as positive for producing healthy food, enhancing yield, and increasing income, the majority did not consider biologicals as safer than conventional pesticides and fertilizers. Most farmers identified that there are potential risks of biologicals. Efforts are needed to create more awareness, improve knowledge and demonstrate practical application of biologicals among smallholder farmers. This is likely to require the engagement of multiple stakeholders from both industry and government as well as agro-dealers.

## CRediT authorship contribution statement

**Tewodros Mulugeta:** Writing – review & editing, Writing – original draft, Visualization, Validation, Supervision, Software, Resources, Project administration, Methodology, Investigation, Funding acquisition, Formal analysis, Data curation, Conceptualization. **Mesia Ilomo:** Writing – review & editing, Methodology, Conceptualization. **Allan Mueke:** Writing – review & editing, Methodology, Conceptualization. **Cecillia Onyango:** Writing – review & editing, Methodology, Conceptualization. **Lerato Matsaunyane:** Writing – review & editing, Methodology, Investigation, Funding acquisition, Conceptualization. **Quenton Kritzinger:** Writing – review & editing, Methodology, Investigation, Conceptualization. **Erik Alexandersson:** Writing – review & editing, Supervision, Resources, Project administration, Methodology, Investigation, Funding acquisition, Data curation, Conceptualization.

## Declaration of competing interest

The authors declare that they have no known competing financial interests or personal relationships that could have appeared to influence the work reported in this paper.
