# Supplementary material for: Smallholder farmers’ knowledge, attitudes, and practices (KAP) regarding agricultural inputs with a focus on agricultural biologicals
Source: Heliyon. 2024 Feb 20;10(4):e26719. doi: 10.1016/j.heliyon.2024.e26719 (PMC10906418; doi:10.1016/j.heliyon.2024.e26719)
Supplement: Multimedia component 1 [file mmc1.pdf]

Agricultural biologicals: Identifying hurdles of use and a Knowledge, Attitude and Practice (KAP) analysis of stakeholders in sub-Saharan Africa

Farmers Questionnaire

Respondents consent form

Title of research: Agricultural biologicals: Knowledge, Practices, and Attitudes in sub-Saharan Africa

I confirm that the researcher/interviewer has explained to me about the research and all items of this consent form. Also and based on the explanation done by the researcher/interviewer, I know that participation in this research is voluntary and that I am not obliged to respond to all the questions in this research. I can also drop from this research any time I consider appropriate. I am informed on the use, benefits, and loses/disadvantages (if any) of this research including my readiness to set aside between 30 and 60 minutes for the interview.

As an interviewee, I confirm the following;

I permit recoding of this interview/conversation	☐ YES ☐ NO
I permit taking steal pictures (photographing)	☐ YES ☐ NO
I permit taking steal pictures of the surrounding environment	☐ YES ☐ NO
My name can be used for purpose of this interview	☐ YES ☐ NO
I allow quoting part of the conversation as found appropriate	☐ YES ☐ NO
I am ready for extended interviews that can be arranged next time	☐ YES ☐ NO

I understand that there are no direct benefits from this research. It may however increase the understanding on biologicals among smallholders farming in SSA. This conversation is for the purpose of research and the researcher/interviewer promise to observe the principle of confidentiality and project interviewee’s information.

Interviewer Name: ______________________________
Interview Date: ______________________________
Mode of interview : face-to-face

1. General information

1.1. City __________________

1.2. Age ___________________

1.3. Gender ________________

1.4. Education level

☐No schooling ☐ Primary education ☐ Secondary education

☐ College training (Certificate, diploma) ☐ Bachelor degree and above

1.5. Livelihood ☐ Farmer (self-employed) ☐ Employed

Others, specify

1.6. Marital status _______________

1.7. Household size : ________ Females_______ Males _________

1.8. Head of household ☐ Man ☐ Woman

1.9. Household annual income in Birr : __________________

2. Agriculture and farming practice

2.1. How long have you been farming (in years)? __________________

2.2. What is your main crop based on the season?

Main rainy season: ______________________________

Off season/irrigation: ____________________________

2.3. What are your roles in agriculture?

☐ Farming ☐ Processing ☐ Input supply ☐ Exportation

2.4. Specify the size of the farm/plot and source of land

☐ Own :_____________ ☐ Hire : _________________

2.5. Which source of labour did you use last season? (Multiple responses are possible)

☐ Family labour ☐ Paid labour ☐ Relative labour ☐ Friends labour

2.6. What was your average harvest volume for the main crop last season? _____ bags/ quintals/tones

2.7. Do you sell your produce? ☐Yes ☐No

2.8. If yes, where do you sell your produce? ☐Local Market ☐ Export market

2.9. If you sell in the export market

To which countries do you export?______________, _______________

When did you start exporting?________________________________

2.10. Are you an organic farmer? ☐ YES ☐ NO ☐ I do not know

3. Agricultural inputs utilization

3.1. Do you use agricultural inputs in crop farming? ☐ YES ☐ NO

3.2. If no, why not?____

3.3. Which agricultural inputs do you use in farming? [multiple responses are possible]

☐ Fertilizers ☐Quality seeds ☐Fungicides ☐Insecticides ☐Nematacides

Other (specify) ____

3.4. Who advised you to use pesticides?

☐ it was my decision ☐ Neighbor's advise ☐ Extension officers advise ☐ Dealers

3.5. Who advised you to use fertilizer?
☐ it was my decision ☐ Neighbor's advise ☐ Extension officers advise ☐ Dealers

3.6. For what purpose do you use pesticides?
☐ To reduce crop loss ☐ To fulfill market demand ☐ others are using

Other, specify: ____

3.7. For what purpose do you use fertilizer?
☐ To improve production ☐ To fulfill gov't order ☐ others are using

Other, specify: ____

3.8. Do you always know which type of pesticide to buy? ☐ Yes ☐ No

3.9. How do you select pesticides? (multiple responses are possible)
☐ I ask a neighbor ☐ I ask a dealer ☐ I ask an extension officer

☐ I just ask for a pesticide I know ☐ I take any pesticide I find

3.10. Do you know the contents of the pesticides or fertilizers? ☐ Yes ☐ No
If yes, specify: ____
3.11. Have you ever had any training on pesticide sourcing and utilization? ☐ Yes ☐ No
3.12. If yes, how was it useful? ____
3.13. Have you ever been advised to use a specific pesticide by someone before? ☐ Yes ☐ No
3.14. If yes, what was the reason?____
3.15. Have you ever been advised **not** to use a specific pesticide by anyone before? ☐ Yes ☐ No
3.16. If yes, what was the reason?____
3.17. In your opinion, do you think pesticides have a negative impact on:

	Yes	No	I do not know
Human health?			
Animal health?			
Environment?			
Crop production?			

3.18. Have you ever stopped using pesticides because of their negative impacts?
☐ Yes ☐ No ☐ Sometimes

3.19. What do you do to minimize the potential impact of pesticides on humans, animals, and the environment?
☐ I use personal protective equipment ☐ I buy safer pesticides
☐ I do nothing ☐ I seek advice from extension officers
☐ I do not know how to minimize the impact

4. Safer alternatives/Biologicals

4.1. Do you know that there are safer alternatives to chemical pesticides?
☐ Yes ☐ No If no go to No 4.7.

4.2. If yes, how did you hear about their presence?
☐Dealers ☐Neighbor/colleague ☐Extension officers ☐Newspapers,
☐Magazines ☐Social media ☐TV/radio ☐Through training sessions

4.3. Have you ever bought a pesticide that is claimed to be safer for your health, your animals' health and / or the environment? ☐ Yes ☐ No

4.4. If yes, please name the pesticide/s you bought?
____

4.5. If yes, Who often applies them in your family the field?_______________

	Man	Woman	Woman and man	Children	All members
1	☐	☐	☐	☐	☐
2	☐	☐	☐	☐	☐

4.6. If you have never bought safer pesticides, why not?

☐ They are not available in the market ☐ They are expensive
☐ It is difficult to store/manage them

4.7. According to your understanding, what are biologicals?
____

4.8. Do you use biologicals? ☐ Yes ☐ No

4.9. If no, why not?
☐Availability ☐Cost(expensive) ☐Access (difficult to get them)
☐Low benefits (low yield) ☐Not aware of their existence

4.10. If yes, where do you buy them
☐Within the village ☐Nearby village ☐At district center ☐At regional center

4.11. Which kind of biologicals did you use in the last seasons? State the names or show the cover or picture of box.

4.12. When did you start using biologicals?_____________________________________

4.13. Did you use biologicals in the last 2 farming seasons? ☐ Yes ☐ No

4.14. Who often buys biologicals or safer alternatives?
☐Husband ☐ Wife ☐ Children ☐ Non-family member

4.15. How often do you use biologicals?
☐Every season ☐Some seasons ☐Seldom (e.g. once in life)

4.16. If you seldom or rarely use biologicals, what could be the possible reason?
☐Expensive ☐Access ☐Low efficacy ☐I am not sure how to use it

4.17. How many times do you use biologicals in a given season? (add rows and specific names)

☐Once ☐Twice ☐More than twice

4.18. Which challenges do you face in using biologicals (rank them starting with the most critical challenges, which takes a value 1)

	Challenge	Rank
A	Lack of knowledge in using biologicals	
B	Preservation/storage challenge	
C	Lack of support (after sale) services	
D	Absence of supplementary inputs (ingredients)	

4.19. Do you remember any time biologicals were not effective? ☐ Yes ☐ No

4.20. Please, name the biologicals that were not effective: _____________, ________________

4.21. In case you have to choose between two types of inputs, which do you often take?

Conventional synthetic inputs	Biologicals	Any of them (conventional or biologicals)	Neither of the two (I have alternative inputs)
☐	☐	☐	☐

4.22. If anyone tells you about the safer alternatives would you consider utilizing them?
☐Yes ☐No ☐I am not sure

4.23. Have you ever participated in training on biologicals? ☐ Yes ☐ No

4.24. Did your spouse participate in trainings on biologicals?
☐Yes ☐No ☐I do not know ☐Not applicable

5. Attitude

This is how I view biologicals and conventional inputs in farming (select what best correspond with your view/perception, single response is expected for each statement)

		Strongly disagree	Disagree	Undecided (not sure)	Agree	Strongly agree
		1	2	3	4	5
A	It is advantageous to use biologicals	☐	☐	☐	☐	☐
B	Biologicals can produce healthy food	☐	☐	☐	☐	☐
C	Biologicals increase yield of crops	☐	☐	☐	☐	☐
D	Biologicals can increase income for farmers	☐	☐	☐	☐	☐
E	Information on biologicals and how to use them is easily available	☐	☐	☐	☐	☐
F	Biologicals are risk free	☐	☐	☐	☐	☐

		Strongly disagree	Disagree	Undecided (not sure)	Agree	Strongly agree
		1	2	3	4	5
G	All biologicals are safe for environment	☐	☐	☐	☐	☐
H	Preferences in the food market influence the use of biologicals	☐	☐	☐	☐	☐
I	There are no conditions or rules in using biologicals	☐	☐	☐	☐	☐
J	Biologicals are easier to use than conventional inputs	☐	☐	☐	☐	☐
K	Biologicals are more accessible (I spend less time, distance to get them)	☐	☐	☐	☐	☐
L	Biologicals are cheaper	☐	☐	☐	☐	☐
M	The community supports the use of biologicals	☐	☐	☐	☐	☐
N	The government supports the use of biologicals	☐	☐	☐	☐	☐
O	I will recommend biologicals as a safer alternative to neighbours/others	☐	☐	☐	☐	☐

Thank you for your time
